# Collectanea

**Published:** 1831-05

**Authors:** 


### COLLECTANEA.

Floriferis ut apes in saltibus omnia libant,
Omnia nos, itidem, depascimur aurea dicta.

### ANATOMY.

*On the Phenomenon of Blushing.* M. E. A. Lauth observes, that he is not aware that any precise information has been afforded as to the kind of vessels which produce the colour of the face. Most physiologists merely say that it depends upon the capillaries. M. L. states that, if the arteries are successfully injected, the whole of the face becomes of an uniform red tint. It cannot, therefore, be these vessels which produce the phenomenon of blushing. He has derived the following results from a perfect injection of the facial veins: the cheeks were deeply coloured; the chin, the tip of the nose, and the forehead, obtained a slighter tint; and the other parts of the face were still less coloured. This kind of coloration resembles that which is produced by mental emotions during life, and we may, therefore, conclude that blushing depends, in part, upon venous congestion.—*Mem. de la Société d'Histoire Naturelle de Strasbourg.*

### PHYSIOLOGY.

*Identity of the Nervous and Electric Fluids.* By Dr. David. (Extract from an Inaugural Thesis, Paris, 1830.)

M. David, having been long struck with the numerous analogies which had been detected between the phenomena of electricity and the nervous power, instituted a series of experiments upon this very interesting question; and the results very strongly confirm those which Dr. Beraudi made, and which we published.* We take the following extract from the Archives Génerales.

In September 1829, M. David exposed the nerves of the thigh of a chicken. He then divided them, and introduced into the neurilema a small brass wire, proportioned to the size of the nerve: the wire was made to touch the nervous pulp. Having then placed, near the opposite end of the metallic thread, a small needle, the latter exhibited very manifest oscillations. These oscillations appeared to M. David to be in proportion to the movements of the animal; so that they were much greater when the motions of the animal were more powerful. At some moments the oscillations of the needle were not manifest, and it was found that then the wire was not in immediate contact with the nervous pulp.

M. David repeated this experiment several times upon the largest nerves, and he frequently found the extremity of the needle describe an arc of four or five lines, and even more. If he forced the animal to struggle, at the same time that he held the extremity of the wire at a greater or lesser distance from the needle, he could evidently prolong the oscillations of the latter.

These experiments were too imperfect to convince M. D. that the nerves transmitted an electric current. The effects might possibly arise from an involuntary shock produced by the wire against so moveable an apparatus: and, again, if the oscillation of the needle did really depend upon an electric current, the latter might be the result of oxydation of the metal. To avoid these probable sources of error, M. D. afterwards experimented with the multiplicator of Schweiger and threads of platina: the results were still the same.

The sciatic nerve of a rabbit was insulated and laid bare, and carefully sponged; a piece of glass was gently introduced between the nerves and the muscles, while the leg of the animal was bent. The sensibility of the nerve was shewn by the motions of the animal during the introduction of the needles, the one above the other, but not touching each other. They were placed in communication with the galvanometer: the animal was quite tranquil, and the needle of the multiplicator at rest. By a sudden movement of the rabbit, the apparatus was deranged, but the needle clearly deviated and moved. The needles were again introduced; some muscular contractions succeeded; again the needle oscillated, but so slightly as not to convince the assistants. The animal, how-

* London Med. and Phys. Journal, November 1829, p. 457.

ever, soon made some very vigorous and repeated exertions, and there was no longer any doubt of the fact, for the needle now described an arc of more than two lines. The oscillations ceased with the motions of the animal, and again appeared when it moved. The animal was excited to make contractile efforts, by stimulating the nostril or irritating the nerve, and the needle immediately oscillated, and the arc it described was great in proportion to the energy of the muscular exertions which were provoked. The phenomena could, in fact, be caused at will. With four needles, double the effect could be produced than when two only were employed. In general, the intensity of the phenomena diminished with the vigor of the animal, and they were not observable after death. When two needles were placed in a nerve, and two in a muscle, the oscillations were barely perceptible. When all four were introduced into a muscle, M. David could obtain no deviation of the galvanometric needle.

Other experiments demonstrated the reason why, sometimes, the phenomena may not arise when needles were placed in a nerve. The causes of the non-occurrence of the phenomena may be either, 1st, insensibility of the nerve from its being strained, or pressed upon, in sponging it; 2d, its too great tension over the glass placed beneath; 3d, blood may cover both the nerve and needles; 4th, the perfect dryness of the nerve produced by the sponge. It is then necessary to place the nerve for a moment in contact with the muscles, and its power is restored. It is highly important that the needles and the extremities of the threads of the galvanometer should be perfectly clean.

M. David considers these experiments sufficient, 1st, to prove that organised beings have a special apparatus, which is destined to furnish an electric current; and, 2d, to shew the circumstances which are required for its production. Needles implanted in a nerve which is completely separated from the spinal marrow, produce no motion in the magnetic needle: but if a nerve is experimented upon *above* the part where it has been divided from the nervous centre, an electric current will be produced, which will cause the needle to deviate. When the spinal marrow is divided between the occiput and the first cervical vertebræ, none of the nerves will give rise to any electric phenomena. Among some sufficiently obvious inferences that M. David draws from his experiments, he observes, that muscular contraction is not produced by an inherent force in the muscular fibre, either irritability or contractility, which are purely imaginary faculties, but by the electric currents furnished by the nervous branches which the muscles receive.

### PATHOLOGY.

*Tympanitis of the Intestines, relieved by the Introduction of a Tube.* By Dr. Touzet. (*Compte rendu de la Soc. R. Med. &c. de Toulouse*, 1829.)

A man, forty-eight years of age, of a nervous temperament, after having eaten an enormous quantity of bread and raw beans, experienced very severe pain in the right hypochondriac region. These pains increased at intervals, and were accompanied by great prostration of strength and paleness of face. Leeches, hip baths, sulphate of soda, and iced water, were employed, without advantage. There was great tension of the belly, and, if the patient felt relief for a moment, it appeared to depend upon frequent eructations. Under these circumstances, and when the symptoms had reached such a degree of severity that the life of the patient appeared in danger, M. Touzet had recourse to the following expedient: he first administered an enema, and then introduced into the anus a hollow tube, about four lines in diameter, compressing, at the same time, the most distended and painful parts of the abdomen. An abundant quantity of air was thus expelled, and the patient was greatly relieved. The tube was retained in its situation by an appropriate apparatus, and compression was kept up upon the belly by a bandage and cushion: gas was thus freely evacuated. The next day, the patient was free from pain. Cordials were now administered with prudence, and a strengthening regimen quickly restored him to perfect health.

---

*Case of Ossification of the Heart.* By James Richardson, Surgeon, Glasgow.

W. M'K., aged forty-eight, porter. Had complained for many months of swelling and pain in the epigastric region, extending into the right hypochondrium, which symptoms were supposed to arise from an enlargement of the liver. He came under my care on 29th September, 1829, at which time the whole abdomen was much swollen, and fluctuation distinct; he complained also of lancinating pain immediately below the ensiform cartilage, and in the region of the liver, but, from the great accumulation of fluid, no distinct tumor was perceptible. Pulse regular, seldom above seventy; urine scanty and high coloured; skin dry, great thirst and deficient appetite. He used squills, digitalis, decoction of broom, and other diuretics, and the system was kept under the influence of mercury for some weeks. By these means the lancinating pains were much alleviated, but the dropsical symptoms increased; the abdomen became larger, the lower extremities œdematous, and his breathing was so much oppressed that it was necessary to perform paracentesis. On the 16th November, sixteen pounds of fluid were drawn off; it was very thick and gelatinous, and had floating in it a great many pieces of coagulated lymph. A large tumor was now discovered occupying both hypochondria, and also the epigastric and umbilical regions. Pressure upon it produced acute pain, particularly towards the right side, and, when this was applied over the ensiform cartilage, a feeling of nausea was induced with dragging pain at stomach.

After the operation, the symptoms were very greatly relieved. He passed urine freely, and expressed himself as being quite well. The fluid, however, again accumulated rapidly, and on the 18th December the same quantity was drawn off; two days after which, symptoms of peritonitis came on, and he died on 28th December.

On dissection, the tumor was found to arise from a scirrhous enlargement of the omentum, which had formed adhesions to all the neighbouring parts, particularly to the stomach and transverse arch of the colon, the last of which was imbedded in its substance, and so much compressed that its canal was nearly obliterated. The liver was not larger than natural, but that part of its left lobe which was in intimate connexion with the diseased mass, had assumed somewhat of the same appearance. There was a great quantity of fluid in the cavity of the abdomen, similar to what had been drawn off. The lungs exhibited no marks of disease. On examination of the heart, the pericardium was found firmly adherent to its right side. When dissected off, nearly the whole of the right auricle, and fully one half of the corresponding ventricle were found invested with a thick and rugged deposition of ossific matter. An osseous lamella, half an inch in breadth, nearly surrounded the heart, following the course of junction of the auricles and ventricles. The left ventricle was marked by numerous striæ of bone, corresponding with the tract of the coronary vessels; and on its upper and lateral surface, an irregular plate of bone, an inch and a half long by three fourths of an inch broad, was deposited. The surface of this plate was smooth, and the pericardium did not adhere to it.

This case is rather singular, from the fact that the pulse was never in the slightest degree irregular; neither was it at any time, prior to the peritoneal inflammation, very quick. It generally ranged between seventy and eighty, and was by no means, even to the last, either small or feeble. There was no symptom of irregularity in the circulation of the lungs, no dyspnœa nor cough. Under these circumstances, no suspicion existed during life of any affection of the heart, and, an examination with the stethoscope not being indicated, that instrument was not employed.—*Glasgow Med. Journal.*

---

*Cases of Idiopathic Glossitis.* By John Orgill, Surgeon, Stranraer.

True idiophatic glossitis seems to be a very rare disease; at least, the recorded cases are few in number.

The following cases, therefore, occurring in my practice here, may not be altogether destitute of importance.

Case I. C. Kenmuire, a farmer, aged fifty, complained of much difficulty in deglutition, which he attributed to inflammation of the throat. As he lived at some distance in the country, and could not come in himself, his wife came to me, and explained the symptoms as well as she could. Appropiate remedies for the supposed disease, inflammation of the throat, were recommended. About a week after this, he was brought to town in a cart. I then found that the left half of the tongue was so much swollen, as completely to prevent articulation and deglutition. The right half, of its natural size and appearance, was in part overlapped by the diseased half. For eight days preceding, he had not been able to swallow any thing solid; and during the last two days, he could not get down a drop of liquid. The pulse was nearly natural. I wished to apply leeches to the tongue; but the mouth was so completely filled with it, as not to afford space for them except at the tip. I therefore put eleven large leeches to the root of the tongue externally; and, when they fell off, applied a cupping glass over the bites, by which means, about six ounces of blood were obtained. But this afforded little relief. I then introduced a scalpel flat on the dorsum of the tongue; and made two incisions about half an inch deep, from the farthest point to which the instrument reached to the tip.

The incisions bled pretty freely; and the swelling was, in consequence, so far reduced as to enable him to answer questions intelligibly. He could also expectorate a little, which he was before unable to do, though, as he expressed it, " choking with his spittle," which was thick and very tenacious.

This was at noon. I saw him again about eight o'clock in the evening. The diseased half of the organ was then as much swollen as ever. I scarified it still more deeply; and ordered an enema with an ounce of castor oil. As he was evidently exhausted from want of food, for which he had a good appetite, but which, as I have stated, he had been unable to take for eight days, I ordered some soup to be made, with the intention of calling in an hour after, and of attempting to introduce it into the stomach by means of the stomach-pump. I accordingly returned, and found him smoking his pipe, the last scarification, along with the enema, having given him great relief. With considerable difficulty, and very slowly, he swallowed a small bowl of soup. When I saw him next morning, the tongue had resumed its former swollen state. I then observed, what I had not done before, a peculiar lividity at the tip of the diseased half of the organ. I now introduced the scalpel as before, and made an incision more than an inch in depth. A great gush of most offensive pus followed, and gave the patient immediate relief. The incisions healed in eight days; the tongue having recovered its proper size and appearance. The sensibility on the left side of the tongue continued impaired for a year after, but it was afterwards gradually recovered.

Case II. March 5th, 1828. James Brown, a sailor, æt. thirtyfive. After languor, and some rigor, complained of difficulty of deglutition, which he attributed to inflammation of throat. I saw him next day, and found the left half of tongue swollen to at least three times its natural bulk, and very painful. Articulation and deglutition were performed with much difficulty and pain. The surface of the tongue was foul, except at the tip, which was peculiarly clean and red. The papillæ of this part seemed to have entirely disappeared, leaving the tip remarkably smooth. The median line formed an abrupt termination of the enlargement. Pulse 100, very hard and full. Some thirst. The abstraction of twenty ounces of blood gave some relief, and enabled him to swallow a brisk cathartic immediately after. The next day, the tumefaction and pain seemed to be again on the increase. Five large leeches were applied to the tongue; and the cathartic was repeated. The leeching gave immediate relief; and, from this time, the disease rapidly abated, leaving the organ in a healthy state on the fourth day after the attack.

Case III. July, 1828. J. B., a woman from the country, applied to me with glossitis affecting the whole organ, and terminating in suppuration of the right half. She was relieved by scarifications, by letting out the pus, by the lancet introduced at the side of the tongue, and by cathartics. Some months after, she was again attacked with the same complaint. As I was hurriedly called away when she came to me, she went to another surgeon, and I never learned the result. In this case, likewise, there was a peculiar lividity, and smoothness at the tip, on the side which suppurated.

*Remarks.* In none of these cases could the patient assign an adequate cause for the complaint, unless we consider as such, the only one that Kenmuire could give. At the first bite of a very sour apple which he had been eating two days before the attack, he felt as if a needle had run in his tongue; and a sudden flow of saliva followed. In the 4th vol. of the Dublin Hospital Reports, a case of idiophatic glossitis affecting the left half of the tongue is related by Dr. Graves; and is, apparently, the only case on record in which the inflammation was limited to the half of the organ. In the first two cases related above, the disease was confined to the left half also. This, of course, must be considered as an accidental coincidence; for we can hardly conceive why the left half should be more liable to inflammation than the right. Perhaps the lividity on the tip, in the first and last cases, may be considered as symptomatic of the suppuration which took place. If so, this would encourage us, in a similar case, to have recourse to incision, as practised in these cases with so much success. I believe it will be found very difficult to detect the presence of pus by the feeling of fluctuation which generally guides us in other cases. The tongue fills the mouth so completely, and the introduction of the fingers gives so much pain, that putting out of the question the unsteadiness of the organ, its peculiar texture, and the deep seat of the pus, it may be considered a matter of importance to fix on some appearance as indicative of the formation of an abscess. So far as these cases go, the livid colour of the tip of the tongue may be considered as symptomatic of suppuration.—*Ibid.*

---

*Absorption of the Iris.* A boy received a blow upon the eye, from a piece of metal, which was followed by considerable pain and iuflammation, and laceration of the iris; for a time, the pupil was cordiform, being pointed at its lower part; slowly and without any pain, the whole of the iris was absorbed, and the eye became amaurotic. I have several times noticed a similar occurrence, namely, laceration of the iris, from local injury, followed by its partial, and in one instance its total, absorption; and in every case the organ so injured has, eventually, become amaurotic.—Mr. MIDDLEMORE, *Midland Med. Rep.*

---

*Growth from the Ciliary Ligament.* A child, ten years old, was struck upon the eye, with the closed hand, by one of his companions, but without occasioning a sufficient degree of inflammation to excite the attention of his friends; in about a week, a small reddish tumor formed in the anterior chamber, at the anterior side of the eye, which appeared only to be attached to the ciliary ligament; it soon became as large as the half of a small pea; was convex anteriorly, and extended nearly across the pupil; it occasioned no pain, and was not accompanied by any perceptible inflammation; the motions of the iris were not impeded, nor was its colour changed; the pupil, also, preserved its circular form. I placed this boy's constitution under the influence of mercury, and was pleased to find that the tumor gradually became absorbed, and did not again return.

I have said this tumor arose from the ciliary ligament, because its attachment was in the situation of that part, and, also, because the action and colour of the iris were unaltered. In the few instances in which I have seen similar growths from the iris, the colour of that membrane has been changed, its action interfered with, and the circularity of the pupil has also been destroyed.—*Ibid.*

---

### PRACTICAL MEDICINE.

*Syphilis cured by Mercurial Pediluvia.* Dr. TAMBONE has cured, by this means, twelve patients affected with inveterate syphilis. In one case, in which the disease had existed for four years, and was complicated with exostoses and ulceration of the glands of the neck, and great emaciation, thirty pediluvia effected a cure. In another, the patient was affected with atrophy of the arm and left leg, anchylosis of the knee, pains in the bones, and ulcers of the palate, with hectic fever: he had, in vain, been treated by the ordinary methods, and was completely cured by forty-nine mercurial foot-baths.—*Osservatore Medico,* Feb. 15, 1830.

### SURGERY.

*Treatment of Syphilis by Cinnabar Fumigations.* By Dr. Werneck. (*Journ. für Chir. und Augenheilkunde*, t. xiv.)

The employment of cinnabar fumigations was common among the ancients, but in modern times it has been abandoned, on account of the accidents which resulted from their improper application. These fumigations, Dr. W. assures us, are very efficacious in cases of syphilitic ulcers of the skin, nose, and nasal fossa, especially when mercury has been given internally without benefit: but it is necessary that the patient should undergo some preparation. Mr. W. commences by a strong purgative, a warm bath daily, for six days, and moderate diet. The patient must not quit his chamber, which must be constantly at the temperature of 14° R. If there are many ulcers, they must be dressed with simple water; and the preparative course must terminate, as it begun, with a purgative. After these preliminary measures, the fumigations may be commenced. The patient, covered with a cloak of waxed cloth, is to be placed upon a seat, under which is the apparatus, which consists of a spirit lamp, and a china plate, on which the cinnabar is put. The cloak must be closely drawn round the neck, that the mercurial vapours may not too freely escape. The chamber should now be at 18° R. Each fumigation lasts about a quarter of an hour, and the patient should retire to bed directly afterwards: M. W., therefore, always applies the remedy in the evening.

When there are any traces of syphilis on the head, or ulcers in the neck or throat, the head of the patient should be surrounded by the cloak. The inspiration of the metallic vapours quickly produces salivation, and, when this symptom occurs, the fumigations are suspended altogether, or the quantity of cinnabar is lessened. (The ordinary quantity is from twenty to forty grains for each fumigation.) In most cases, eighteen or twenty fumigations complete the cure; one daily is sufficient. In some rare instances they cannot be used oftener than every two or three days.

During this treatment, it is to no purpose that the patient changes his linen, as it is constantly soiled by mercurial particles. When the treatment is completed, a soap bath must be used, and the patient must remain in his chamber for fifteen days, and abstain for some time from all stimulating liquors.

At the end of his memoir, the author gives the result of this practice in eighteen cases. The longest treatment was fifty-eight days; the shortest, seventeen days.

---

### MIDWIFERY.

*Prolapsus and Rupture of the Uterus during Labour: recovery.* A woman, thirty years of age, of very weak constitution, and the mother of several children, had had prolapsus uteri since her last labour; to which, however, no attention had been paid. She again became pregnant, and the first pains of labour began towards the end of the eighth month of utero-gestation. The labour was at first regular, but; after some hours' duration, the inferior segment of the uterus passed out of the vulva, and fell between the thighs, where it appeared like a cylinder, six inches long and two broad, terminated at the lower part by an orifice with thick edges. Upon the surface of the protruded part, several livid spots were seen. The head of the child presented at the vulva, and the uterine contractions, which were powerful, must quickly have expelled it, if the obstacle caused by the protruded part of the uterus had not been too great to be surmounted. Dr. Henschel applied the forceps, and drew the head of the child to the os uteri; but, at that moment, a laceration took place of the upper part of the protruded portion of the womb. The forceps were withdrawn, and the head of the child was soon expelled: it lived but a short time.

The placenta having come away, the inferior segment of the uterus contracted in length, but its bulk was much increased. The uterus was returned as soon as possible. The rupture had taken place just above the cervix, and was at first about half an inch long; but it was much diminished when the organ contracted, and after a few minutes was no longer perceptible. Some severe symptoms arose, but they soon disappeared. For a short time the lochial discharge was mixed with pus. On the tenth day, the patient was able to leave her bed, and in a month she was in perfect health.—*Siebold's Journ. für Geburtshülfe*, t. viii. p. 717.

---

*On the Use of the Secale Cornutum.* Dr. Armour, who has recently published an interesting paper on this subject, in the Glasgow Medical Journal, quotes the following results obtained by Villeneuve, in 720 cases.

"1st. In 600, the success was complete in cases of labour properly so called, that is, for the expulsion of the fœtus alone, living or dead, at the term or otherwise; the pregnancy being simple or of twins.

"2d. Five cases of success, in the expulsion of the placenta.

"3d. Five cases of success, in flooding, after delivering.

"4th. Sixteen cases of incomplete success, which consisted 1st, of cases in which the ergot only excited the expulsive powers, for a certain time; the delivery not being terminated naturally, till several hours after the employment of this medicine: 2dly, of cases in which, after having advanced the labour to such a degree, as to render the application of instruments possible, these were employed.

"5th. Eighty-two cases of complete failure, or cases in which the ergot had no sensible effect; or, if you will, did not produce any return of uterine contractions, in whatever doses it was given.

"6th. Twelve disagreeable or fatal results, either for the mother or the child, attributed by different authors, either to the immediate action, or to the secondary effects of the ergot.

"It follows, that of 720 cases, in which the ergot has been employed, 610 have been complete, not reckoning those of incomplete success. This is nearly seven and a half of success to one of failure, a result whieh is seldom furnished by other therapeutical agents employed to combat any morbid state, the cessation of which, as well as the favorable termination of labour may, in many cases, depend solely upon the resources of nature alone."

### MISCELLANEOUS.

*Singular Result from the external Use of Corrosive Sublimate.**—A gentleman had been troubled with "certain disagreeable animals," and was directed to rub in, on the part affected, an ointment of white precipitate and spermaceti cerate. He was by this means relieved from his troublesome companions; but, as he was visited by another party of them, he was again obliged to resort to their destruction. He now procured, by mistake, a small packet of corrosive sublimate: five grains of this he reduced to powder, united with salt butter, and he rubbed the mass briskly in over the whole of the lower part of the abdomen, the penis (not the glans), the scrotum, and the perineum. From this application he suffered very severely, but nothing was done for one or two hours, and then, for a long period, cold water and flour were applied, to assuage his torment. Next morning the pain was lessened, and shortly after a tingling sensation only remained. The entire cuticle of the scrotum desquamated, having first risen all over in small blisters, filled with a pale yellow, barely fluid, pus. The torment was most severe in the testes: these appeared to be consuming by exposure to fierce flame. No further symptom followed; but, seven days after the mistake, when trying to polish the ring on his hand with one of his fingers, he was astonished at discovering an appearance of mercury on the gold, and, proceeding to burnish the metal all over, he readily covered the entire surface with a plating of quicksilver. The circumstance was made known to a medical gentleman, and the discs of three sovereigns were also mercurialised.

The following morning the relator of the case saw the party, and by rubbing the handle of a gold eye-glass upon the inner surface of the arm, a similar result was obtained. A portion of the milled edge of a sovereign was also thus so completely coated with mercury, that no glimpse of the gold could be seen through it. The mouth was strictly examined, but not the slightest ptyalism, enlargement, unusual redness, or looseness of the teeth, was discernible, or had for a moment been experienced. The health was as usual; there had been no exposure to cold air; diet had been moderate, with large quantities of warm diluent fluid; personal appearance unaltered.

* This curious case was lately published anonymously in the Lancet. The name and address of the relator of it was given to the editor of that Journal that no doubt might be entertained as to the facts.

### ROYAL COLLEGE OF SURGEONS.

### *Introductory Lecture, delivered by* G. J. Guthrie, F.R.S. Professor of Anatomy and Surgery to the College.

My object, on the present occasion, is to notice certain points connected with the lectures delivered at the College, and with the progress and present state of the study of anatomy and surgery, which I omitted in the last Introductory Lecture.

In the year 1646, Mr. Edward Arris, alderman of London, and master of the corporation of barber surgeons, founded the first six lectures, by giving £300., and subsequently £210. for this purpose; and his example was followed by Gale, in 1698, who added to the sum left by Arris £16. a year, charged upon houses in Snow hill, and which was bought up by the corporation of London, in 1806, for £432. At this early period, the advantages to be derived from public instruction were acknowledged, and on the endowment of this College, by the gift of the Hunterian collection, twenty-four lectures were added, making a total of thirty; half to be delivered by the professor of human anatomy and surgery, and the remaining portion by the professor more particularly restricted to comparative anatomy. This College has also given annually two prizes, of small value it is true, but still sufficient to excite the emulation of many able men, and several excellent anatomical and surgical works have been the result. It has latterly, in the instance of a very laborious work on the Nerves, by Mr. Swan, offered to take upon itself the expense of the publication of the engravings: the offer was declined for reasons with which I am unacquainted; although it is to me a matter of great regret that it was not accepted, because it was one of the greatest marks of distinction which this College could bestow; and I can only hope that another opportunity will soon occur, that it will not be long before another author will present himself, whose labours may be deserving of so signal a mark of approbation; and I shall feel individually happy, and I am sure every member of the council unites with me in this feeling, in being the proposer of such an honorary reward, the highest, in my opinion, which this council can give to one of its deserving members.

Since the period that this body received the honourable appellation of a College, the support and maintenance of the study of anatomy have been the constant care of the court of examiners and council. It is possible they may have rendered themselves open to the charge of having overlooked the means to obtain the end; but if they have done this, it is a fault, or an error, which is common to the profession at large in the anxiety entertained for the diffusion of knowledge, as I shall presently have occasion to notice. During the war, the demand for qualified surgeons was so great, that the court of examiners found it necessary to confine their examinations within much narrower limits than they do at present; yet persons competent to hold the diploma could not be found, and, in the army, it was thought advisable to seek assistance from persons of inferior ability, who obtained only a warrant, instead of a commission. The mischief, the destruction committed by many of these persons was quite dreadful; lives were sacrificed in all directions in a manner too melancholy to dwell upon; and I can only hope that, if the country should again engage in war, so horrible a system will not be had recourse to, but that qualified men only will be employed; and, like every thing else, I have no doubt they will be readily obtained, if the remuneration be equal to the demand.

When peace admitted of a revision of their proceedings, this council, desirous of enforcing the cultivation of anatomy, insisted on a certain length of time being spent in the study of it in London; and in order, at the same time, to procure the best instruction for their students, the teaching of it was restricted to the existing schools, and to such as might be formed in connexion with the recognised hospitals in London. This was certainly a strong measure; it was looking only to the end, without considering the means, and, as it precluded many from attempting to get their bread by their labour and their talent, was assuredly an unjust, although a very beneficial one: for anatomy is not to be taught, or to be learned, by mere dissection alone; the intimate structure of parts must be shewn, by injection, maceration, drying, &c.,—by, indeed, a variety of expensive and laborious processes; and after this has been fully developed, and the functions explained, the changes from this natural structure to the morbid state must be as carefully demonstrated. For this purpose a museum is required, which is a constant source of expense, and can scarcely be formed, unless in large towns, and by the assistance of many persons. The true teaching of anatomy includes, then, physiology and morbid anatomy, without which mere anatomy is comparatively useless, and, to obtain this combined method of teaching, the restriction was useful. It prevented however competition, and was therefore very defective. It could not long exist, because it interfered with the liberty of the subject: it deprived a man of his inherent right to get his bread by the sweat of his brow. It was therefore abandoned, and the teaching of anatomy was thrown open to every one in London. Did individual teachers gain any thing by the removal of the restriction? Nothing but the recovery of the right of teaching, which was almost nothing; it being next to impossible for private teachers to compete with the great establishments, and with their splendid museums, supported by the influence of the persons connected with them. The number of teachers has been scarcely, if at all, increased: nevertheless, the public has gained; the door is open for competition; the drones are necessarily driven from their hives; a teacher is not permitted to occupy himself with several branches of science in succession. The regulations of this College confine him to two at most; and I believe it would be for the advantage of the student if each teacher were limited to one subject.

During the period of restriction, whilst students were obliged to reside in London for nearly two years, many of them qualified themselves to that degree, that they, in turn, wished to teach in their respective hospitals in the country. This was at first refused; but, as the private schools they had formed increased in character, as their collections of natural and diseased structures became augmented, the council felt it would be unjust to continue their restriction, and, in acknowledging the certificates of instruction from provincial schools of anatomy and surgery to be equal with those obtained in London, they also felt the necessity of guarding the public against annoyance or deception. They left the public teaching open to competition in every town; restricting it, however, to such towns only as contained hospitals considered competent for the purposes of instruction. The public has been saved the annoyance of dissecting rooms, where a sufficient and complete course of instruction could not be carried on; the student has been spared the loss of his time and money; whilst the private course of tuition between master and pupil has not been infringed. This council has required that the lectures given in the large towns should be assimilated, both in number and duration of time, to those in London; and teachers only are acknowledged who can bring some proof of their competency; they must, at least, be members of one or other of the legally constituted Colleges of Surgeons of the empire. The whole of the anatomical and surgical lectures required may be attended in the country, and the supply of bodies necessary for these different courses is thus obtained from a number of places, instead of being confined to London alone. The happiest results have followed, and although, under the prejudices against dissection which exist, and the impediments which have been and are thrown in its way, it has not been followed up as might be desired; still the want of it has been much less felt than in former seasons, and I have no hesitation in saying, that a very little legislation would remove all difficulties on the subject.

From this short sketch, it will be seen that the study of anatomy has been maintained, and indeed enforced, in this country, by this College: other public bodies, it is true, may have the right of dissecting the dead; but, with the exception of the College of Physicians, it has been with them a right they have never exercised, and I may assert, that this College has been the main stay and prop of anatomy in England. Without its interference, it would have fallen into disuse: our present able race of surgeons, throughout the country, are indebted to it for the necessity it has inculcated and enforced of studying anatomy; and, if some of its regulations have been severe, they have been useful. This council is, at all times, ready to consider, and even yield to the representations of its members, when they are perceived to be founded on public views alone; and when I look around me, and see that the greatest public men, that those who have been engaged in the legislative affairs of the country, for the whole, I may almost say, of their lives, are frequently obliged to change, and even rescind their measures, I do not think it can be urged as a defect of wisdom in this council, that it has done, and will again do, the same.

In order to render the study of anatomy in this country as perfect as human industry and ability can make it, support is necessary from government, not in the shape of that which has been called "the Anatomy Bill," but by a legislative measure which shall be founded on public principles and views alone; which shall carry its objects and intentions on the face of it; and not, like that most disgraceful Bill, be a thing that "says every thing but what it means, and means every thing but what it says."

In my Introductory Lecture of last year, I pointed out many of the unjust and oppressive claims contained in it: I shall now proceed to notice some others of equal magnitude, and to prove its jesuitical nature. It has been said that this Bill was to be a safeguard to the public against the practices of the profession, and particularly against the proceedings of this College. I shall shew you that it is in the wisdom of this College that the safeguard lies, and not in this Bill, which seems, as I have said, to mean every thing but what it says, or what its advocates say of it. Let us take its first clause: it provides only the same punishment for resurrection men which previously existed; whereas, if the legislature grants a reasonable supply of bodies for dissection, the race should be extirpated: such people should not be allowed to exist. Transportation should be the award of every one engaged in such a traffic. Let me draw your attention for a few moments to the subject of the taxation to be imposed of five guineas on every teacher of anatomy, or surgery, or midwifery: it is not a great sum, but it bears very unequally. Five guineas is an oppressive sum to a beginner, to a private teacher, with his six or ten pupils: it is nothing to a teacher in a large hospital, or public establishment, with his 150 or 200 students. These gentlemen have no objection to it; but, if they were taxed according to their numbers, and had to pay seventy-five or one hundred guineas, we should see them petitioning and remonstrating against the tax, in a manner which could not fail to be effectual. They know that it falls severely on the poor man, or the beginner, who can least afford it; that it acts like a restriction, that it tends to prevent competition, and they do not object to it. I do, because I take the part of the poor against the rich, and because I will not willingly agree to a tax on any scientific pursuit, unless the circumstances of the country render it absolutely necessary.

The tax of two guineas a head, generally, on a practitioner wishing to dissect privately for his instruction, is absolutely monstrous: it is contrary to all sense and reason; it might have been permitted some hundred years ago, but the proposition in the present day is unpardonable. The persons on whom it will fall will be those who, having some operations to do of importance, are desirous to renew their acquaintance with the parts they are to cut. It is said that they will get the body so much cheaper, under the proposed Bill, than they would have done without it, that it compensates for the tax: nothing can be more fallacious. It must be proved that they pay any thing at present for such dissections; and the fact is, they do not. The mischief is not, however, in the two guineas' tax, but in the necessity for having the licence beforehand, which few men will take the trouble to do. I am confident, if the tax be imposed, that not two hundred men out of nine thousand will take out a licence: mark then what will take place. Suppose a surgeon, in any moderate-sized country town, is called upon to perform a serious operation in the course of a few hours. He does not feel quite confident in his knowledge of the parts; he knows there is a dead body in the poorhouse, and he applies to the surgeon or master of it for leave to make the dissection, to look at, and to renew his acquaintance with the subject, before he puts the life of a fellow-creature in jeopardy: he obtains permission, but he has not a licence; he has not paid his two guineas; he can only do it at two or three stated periods of the year; he must give up his dissection, and let his patient die, perhaps kill him if he tries to do the operation, or run the risk of an information, which may be laid by any common informer, and a fine of fifty pounds, which may be his ruin. I repeat that such a tax is monstrous in a civilized country. If it be said, every surgeon must be made to take out a licence, I should like to know what is to become of the money. There are about 150 teachers recognized by this College, who must pay five guineas each, which amounts to 750 guineas. Surely this is enough to pay a secretary, find apartments, and pay stationery and postage, without the two guineas' tax. If that be persisted in, the additional sum can only be raised for purposes of oppression and absolute tyranny, which are hinted at, but not fully expressed in the Bill; and, as it has been for the present withdrawn, I will not take up your time by commenting upon them.

There is another clause, to which I shall now draw your attention: it is a very mild, modest, gentle clause, appearing simply to authorize the commissioners in anatomy to make regulations, and yet it is the most portentous one in the whole Bill; it says nothing, and means every thing. These few unassuming lines were sufficient to set two great public bodies in motion, on opposite sides of the question. The University and College of Surgeons of Dublin protested against it, and I am informed the Irish members of Parliament signified their intentions in so effective a manner, that Ireland was altogether left out of the Bill. The University of Edinburgh, and, I believe, the College of Surgeons, after a due but quiet investigation, gave their assent; and the members for Scotland did not, I understand, object to it. What concealed meaning could there be in this apparently inoffensive clause, that could set two such bodies in opposition, when thoroughly understood by the one, and duly explained to the other? Two words will give you the clue to the labyrinth: "exportation," "importation." The Irish teachers soon found out that it was intended that "the regulations" should extend to the exportation of dead bodies from Ireland to Bristol, Liverpool, Glasgow, &c. &c.; and the Scotch teachers discovered that bodies were to be imported *ad libitum*, for the supply of their schools: in other words, the Irish schools were to be greatly injured, if not totally ruined; and the English provincial and Scotch schools were to be supported at their expense. The decision of the Irish members having put a stop to this as far as regarded Ireland, how was this free trade in dead bodies to go on? Why, London was, of course, to furnish the supply, and the steam boats, having brought up cargoes of salmon, were to carry back, in return, cargoes of cockneys, and drop them, as they went along, at Scarborough, Whitby, Shields, Edinburgh,

Stonehaven, and Aberdeen, as they might have been previously ordered. That political economists should be found absurd enough to desire a free trade in dead bodies, is possible; but such gentlemen seem to forget that the first principle of a free trade is, that the article itself should be free, not subject to restrictions, and particularly to restrictions founded on the prejudices of mankind. Alive or dead, man is never free from restrictions: he cannot marry, but in a particular way, i.e. according to law; he cannot beget, and bring his children into the world, without submitting to the rules and customs of society. He could not, until lately, be buried, unless wrapped in materials prescribed by law, and not then until his death had been duly verified. It is a misdemeanour to disturb his remains, and yet gentlemen suppose they can at once have a free trade in an article so surrounded, so guarded, as it were, not only by the laws, but by the customs of society. The supposition appears to me to be one of those chimeras which even wise people sometimes wander after in vain.

In the Introductory Lecture last season, I stated that the schools of anatomy in Dublin were well supplied, although murderers had been dissected publicly. The editor of the Medical Gazette, in commenting on this remark, was pleased to say, I was mistaken, and that a great commotion had taken place on account of it in Dublin. In this he was in error: he was right as to the fact of the commotion, but wrong as to the cause: it arose from the circumstance of agents having gone over to Dublin, from Scotland and England, to purchase and export dead bodies, to drive in fact a free trade in them. They offered as much as ten pounds a head for them; a price utterly incompatible with the safety of human life in Ireland. The customhouse officers, acting under orders, winked at the packages passing through the customhouse; but the Irish people would not suffer it: Pat had no objection to be exported alive, with his bulls and his pigs, but he had no idea of having his relations exported dead, to be cut up in England and Scotland. Hence commotions took place, which were only suppressed by the practice of exportation being done away with. The Irish government interfered; the customhouse officers ceased to connive at the packages passing through the customhouse; the riots ceased, and I believe no one present or absent will say, that the schools of anatomy in Dublin have not been as well supplied this last winter as ever they were.

The deportation of dead bodies should not be permitted, except in the case of those culprits who have died in prison, or have undergone the last sentence of the law; it is an indignity which should be reserved for them alone. If the body of a poor but honest man must be dissected for the public advantage, it should be done on the spot, that is, in the town in which he died; and students must come to places where the supply is greatest, to obtain instruction. If the inhabitants of great towns will not allow their own dead to be dissected, they have no right to make them schools of anatomy: they are not to become so at the expense of their neighbours. It is an unjust law, which forcibly takes from one town that which it possesses, to give to a neighbouring town, which possesses it in an equal degree in proportion to its numbers, but does not choose to make use of it. If the inhabitants of Bristol, Liverpool, Scarborough, Whitby, Edinburgh, Aberdeen, &c. wish to have schools of anatomy in their respective cities, let them find the means, the dead bodies, necessary to make them so: if they will not do this, let the students go where they can find what they want; but do not make a law, under which one town shall be made to furnish another with that which it possesses, but does not choose to make use of; do not let the feelings of one set of people be outraged, that those of another may be preserved inviolate. There are many ways in which this might be further urged: I will not detain you longer upon the subject, but proceed to the remaining points I intend to notice, and they are, that, under "the regulations," every place might become a school of anatomy, and every man, qualified or otherwise, might become a teacher; yet this is the Bill which has been lauded as the safeguard of the public against the practices of the doctors, and principally of the evil ones of this College! Let us compare our regulations, and the intended regulations of the Bill, in these points. We receive certificates of study only from schools of anatomy established in towns where there are large hospitals, and in which the teachers are qualified men, i.e. are members of one or other of the legally constituted colleges of surgeons of the empire; which is an important restriction. What did the Anatomy Bill propose? that schools might be licensed in every town, however small, in which a person should be found desirous of opening one: in other words, that the legislature should patronize the opening of human shambles in every town in England, whether they could be of use or not; for it is absurd to suppose that the regulations of this College could withstand the provisions of an Act of Parliament. They might be repealed, it is true; but it is a much more easy task to prevent their adoption. This Council, therefore, steadily opposed the measure; by doing which, they considered not only public utility, but public feeling and safety.

The Bill proposed that every man should be the judge of his own qualification to teach anatomy and surgery. It was argued, that, if every man was to be a judge of his own qualification on the important point of teaching, there could be no use, in a college of surgeons or a court of examiners, investigating the capability of a man to practise; and when this, the only point conceded, was yielded, it was done in a manner that must excite surprise, by tacking on to the concession, that the commissioners of the Anatomy Bill should have the right, if they pleased, of appointing a court of examiners to judge of qualification!!! It was on these two points particularly that this council decided on opposing the Bill to the last extremity: they knew they might get the tax altered, but they felt bound towards the public to resist these two points *in limine*. The Bill was thrown out before the College could be heard; but if it appears again, I feel assured they will be equally ready to do their duty.

The medical advocates for this Bill seem to me to look to the end, without regarding the means; and, with all due respect, I cannot help thinking that this has arisen from cupidity, from greediness to have the schools stocked with bodies, which is equally an error; for a dead body will neither be valued nor well dissected, unless it is worth a certain price; and it is better, and will be better for the profession and the public, that dissection should stand as it does at present, than that the profession should be depreciated, and the public outraged, as both must be if the Anatomy Bill were to become a law.

There is one point yet to be noticed: it is as to the nature of the supply of dead bodies. Some medical men, who have had, and still have, the opportunity of writing in the Quarterly and other Reviews, have advocated two points, both of which appear to me to be erroneous, and by no means connected with each other. I have, on two occasions, entered into this subject, but I still cannot abstain from a few observations. The first is, that the dissection of murderers, as a part of their punishment, shall be repealed, and their bodies given to their friends; the second is, that all unclaimed bodies, usually buried by parishes, shall be given up for dissection.

The principal reason given for the first is, that, as long as murderers are dissected as part of their punishment, decent, well-behaved people will not leave their bodies for dissection; but that, if murderers were not dissected, many decent, well-behaved persons would be disposed to leave their bodies to be dissected: that is, dissection would become an unobjectionable process, and might, indeed, become fashionable. Only think, Mr. President, how a beautiful woman might perpetuate the recollection of her beauty, by having her skeleton hung up in this college, or any other part bottled in spirits: it would be another species of immortalization, which our neighbours on the other side of the water, with all the ardour of their imaginations, know nothing about. On a late occasion, the private anatomical teachers of Paris petitioned the authorities for a share of bodies, to enable them to carry on their operations, but they never thought of making dissection either unobjectionable or fashionable, like our reviewing liberals: they did not ask for the bodies of Benjamin Constant and General Foy, that they might immortalise them by handing down their skeletons to posterity; no such thing; their heated imaginations had nothing half so ardent about them; they asked only for the bodies of those who died in prison, or unclaimed in hospitals, and they left the idea of making dissection unobjectionable and fashionable by law, to their more sober brethren on this side the water. They perceived the absurdity of it; they knew that law could do little against the feelings and the prejudices of mankind. What did the laws do that were formerly enacted to regulate so trifling an article as dress: had they any influence? None whatever: and could it be supposed that a law, which enabled a man to sell his own body, and entitled the purchasers to take it, would make dissection unobjectionable, if not fashionable; that the best feelings of mankind were to be overcome by Act of Parliament. It was, and is, an absurdity; and, so far from this part of the punishment of murderers being removed, it ought to be the fate of all persons who are hanged, and of all who die under sentence for a criminal offence.

The advocates for omitting this part of the punishment on murderers, mean something or nothing. In fair discussion, I take it they must mean that it is a stigma, which, when removed, will leave dissection without a taint, and, of course, that it will become unobjectionable. If such be their meaning, why ask for the unclaimed bodies of the poor? why not ask for a proportion of the rich, of all classes of society? The answer is very simple: they may ask, but they would not receive; they may say, dissection is an unobjectionable process, but none believe them; they do not seem to believe it themselves,—at least, if I may judge by their actions. I have already, on another occasion, inquired into, and, I believe, have shewn, the absurdity of calling on medical men to give up their own bodies: I have done this, however, only on the ground that dissection is an objectionable process; but if medical men argue that it ought to be unobjectionable, they are, in my opinion, bound to set the example; but none of them will do it; they will talk, but will not act up to it. The author of the article in the Quarterly Review, a late estimable and learned physician, who laboured hard during his life to make people believe that dissection was an unobjectionable process, did not leave his body for that purpose. His friends and family followed him to the grave, with all the regret and sorrow so good a man could inspire; they mourned over his remains, they have preserved them inviolate, they have set, if you will, an example all good men will follow; they have shewn, in fact, that they do not believe a word the worthy doctor had said or written upon the subject. Now I, who, I believe, am the greatest supporter of the opposite opinion, say to you distinctly, that no one having a family will leave his body for dissection, unless he be a very eccentric person, or a young enthusiast; that no one has a right to inflict on his wife, his children, his friends, the suffering such an act would inevitably cause them, if they felt what they ought towards him. I tell you, I shall not leave my body for dissection; not that I care what becomes of it, but because I do not think I am entitled to do it; and, if I had a SON so lost to all the kindly feelings of our nature, who, I had reason to believe, would give my body up for dissection, I should mark my sense of detestation of his loss of principle, if I had but one shilling, by leaving that shilling away from him.

To conclude, dissection is a process which can only be supported by law. The first objects ought to be those who have died under the odium and punishment of their fellow-citizens, on account of the offences they have committed against them. If these do not suffice, then, and then only, may the law seize upon the bodies of the unclaimed and *unknown*: I say *unknown*, that the law may be equal for the rich and the poor. If a man falls in the street, and dies with a thousand pounds in his pocket, and is unclaimed, his body should go to the dissecting room, his money to the poor. When a poor man dies in any way, and his body is unclaimed and unknown, it should equally go to the dissecting room; but if there be one individual who says he was his friend, and can prove it, then he should be entitled to follow him to the grave, and to mourn over his resting-place, if he pleases, although he should be buried at the public expense. A Bill brought into Parliament on these principles would displease no one, and, if proposed with a due respect and reservation for the rights and ceremonies of the church, would readily pass into a law; whilst it would afford, without distressing the feelings of any individual, an ample supply of bodies for the study of anatomy.

